# miR-29c Increases Protein Synthesis in Skeletal Muscle Independently of AKT/mTOR

**DOI:** 10.3390/ijms23137198

**Published:** 2022-06-28

**Authors:** Paula Ketilly Nascimento Alves, André Cruz, William J. Silva, Siegfried Labeit, Anselmo Sigari Moriscot

**Affiliations:** 1 Department of Anatomy, Institute of Biomedical Sciences, University of Sao Paulo, Sao Paulo 05508-900, Brazil; paulaketilly@usp.br (P.K.N.A.); andrecruz@usp.br (A.C.); williamsilvaj@gmail.com (W.J.S.); 2 DZHK Partner Site Mannheim-Heidelberg, The Institute for Integrative Pathophysiology, Faculty for Clinical Medicine Mannheim, Universitätsmedizin Mannheim, University of Heidelberg, 68169 Mannheim, Germany; labeit@medma.de

**Keywords:** skeletal muscle, mice, AKT, mTOR, miR-29c, protein synthesis

## Abstract

microRNAs negatively regulate gene expression by blocking translation or increasing mRNA degradation. In skeletal muscle, these molecules play important roles in adaptive responses, and ongoing investigations are necessary to understand the fine-tune regulation of skeletal muscle mass. Herein we showed that skeletal muscle overexpression of miR-29c increased fiber size and force at 7 and 30 days after electrotransfer. At both time points, AKT/mTOR pathway components were downregulated, and, surprisingly, overall protein synthesis was strongly elevated at day 7, which normalized by day 30 after pCMVmiR-29c electrotransfer. These results indicate that miR-29c expression induces skeletal muscle hypertrophy and gain of function, which involves increased overall protein synthesis in spite of the deactivation of the AKT/mTOR pathway.

## 1. Introduction

Skeletal muscle tissue is a major component in the body, and its contraction-dedicated cells generate macroscopic movements, allowing for efficient environmental assessment. Accordingly, this tissue also plays a key role in energetic metabolism, nutritional reserve, and thermal homeostasis [1,2,3]. The capacity of skeletal muscle to change its mass and metabolism in feedback to burden and nutritional status provides excellent adaptability to the organism in an ever-shifting environment [1,4,5].

Mechanistically, protein turnover has been identified as a major factor connecting metabolic signaling to mass and fiber size. In an anabolic state induced by plentiful nourishment and/or increased load, the activated mTOR pathway leads to muscle growth by stimulating de novo protein synthesis while negatively regulating the protein breakdown [6,7,8].

More recently, non-coding RNAs have been identified as potential players to modulate cell function in various conditions, such as cell division [9] and embryonic development [10], and, interestingly, in the skeletal muscle field, emerging fast-growing knowledge suggests that non-coding RNAs can play an important role in mass control, making these molecules a promising strategy for developing therapeutics to prevent muscle wasting [9,10].

We have previously shown that miR-29c overexpression can improve skeletal muscle mass and force, which is associated with the downregulation of Atrogin-1, MuRF1, and HDAC4 mRNA and protein levels [11], highlighting this microRNA as a putative therapeutic target in muscle wasting diseases [12]. Although it is clear that miR-29c is involved in skeletal muscle mass gain throughout decreased levels of atrophy-related factors, the impact of this molecule upon anabolic pathways (key processes associated with skeletal mass gain) has not been yet explored, and herein, we addressed whether miR-29c overexpression can impact the key AKT /mTOR pathway and general protein synthesis in skeletal muscle.

Taken together, our results showed that the AKT/mTOR pathway can be nearly entirely inhibited by miR-29c overexpression; nonetheless, overall protein synthesis is robustly increased. These data demonstrate that miR-29c stimulates protein synthesis independently of the AKT/mTOR pathway and provide significant insight into the mechanisms that govern the effects of miR-29c in skeletal muscle.

## 2. Results

### 2.1. GFP Transfection Efficiency and Overexpression of miR-29c Results in Skeletal Muscle

**Figure 1:**
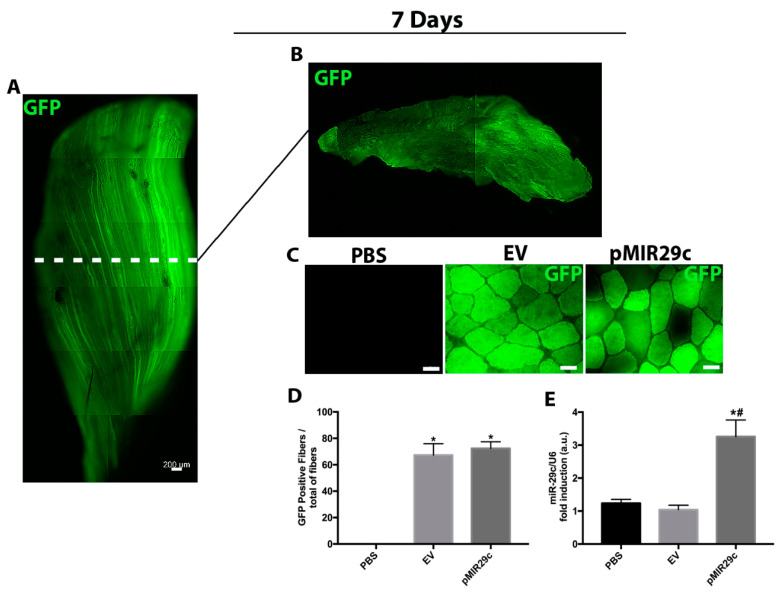
Plasmid transfection efficiency and levels of miR-29c in skeletal muscle. (**A**) Representative photograph composition of tibialis anterior and (**B**) the correspondent transversal section (GFP in green). (**C**) Representative photomicrograph of tibialis anterior muscle cross-section at 7 days of miR-29c overexpression showing positive (green) fibers. (**D**) Efficiency of transfection of the EV and miR-29c expression plasmid 7 days after electrotransfer. (**E**) RNA levels of miR-29c were determined by qPCR in muscles 7 days after electrotransfer. Data were expressed as a percentage of total fibers and arbitrary units (au) as mean and SEM (*n* = 4–5 per group) and were corrected by the endogenous U6 RNA. Statistical analysis included one-way ANOVA followed by Tukey’s posthoc test and the Kruskal–Wallis test followed by Dunn’s posthoc test. * *p* < 0.05 vs. PBS and # *p* < 0.05 vs. EV group.

Firstly, we determined the efficiency of in vivo transfection by measuring the percentage of GFP positive fibers and miR-29c levels by qPCR analysis 7 days after electrotransfer. Consistent with the previous study, we observed GFP widely expressed along the tibialis anterior; about 70% of skeletal muscle fibers were found positive in both EV and pMIR29c groups (Figure 1A–D) [11]. In contrast, no GFP-positive signal was detected in the group only injected with the vehicle (PBS) group (Figure 1C). miR-29c expression was elevated approximately 3-fold at 7 days after electrotransfer when compared to either PBS or EV (Empty Vector) groups (Figure 1E).

### 2.2. Impact of miR-29c Overexpression on Skeletal Muscle Mass and Function

**Figure 2:**
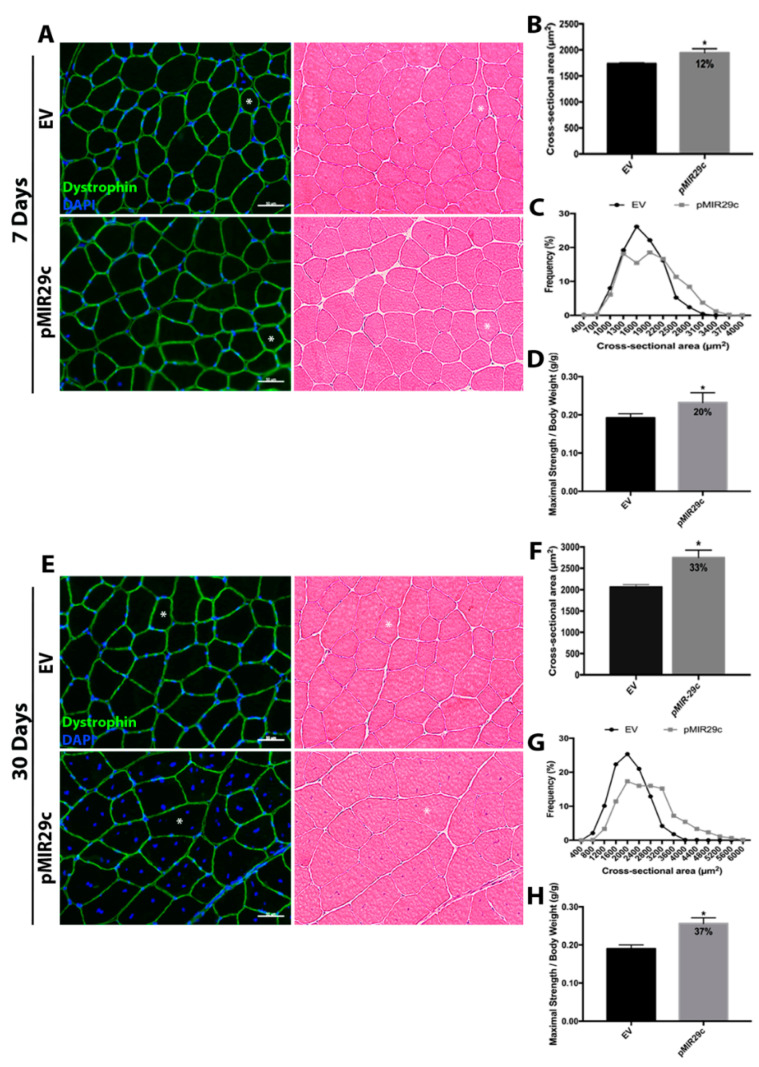
Histological and functional analysis after miR-29c overexpression for 7 and 30 days. (**A**) Representative immunofluorescence photomicrographic (Dystrophin in green and DAPI in blue) and hematoxylin/eosin staining (HE) of tibialis anterior muscle after 7 days and (**E**) 30 days of miR-29c overexpression (scale bar 50 µm, asterisk indicates the same muscle fiber). (**B**) Cross-sectional area of tibialis anterior muscle fibers after 7 and (**F**) 30 days of miR-29c overexpression (*n* = 4–5). (**C**) Frequency distribution of the tibialis anterior muscle fibers by levels of fiber cross-sectional area after 7 and (**G**) 30 days of miR-29c overexpression. (**D**) Maximal strength after 7 and (**H**) 30 days of miR-29c overexpression (*n* = 5). Data were expressed as mean and SEM. Statistical analysis included an unpaired *t*-test. * *p* < 0.05 vs. EV group.

Subsequently, we determined the effects of miR-29c overexpression in skeletal muscle tissue by measuring the skeletal muscle fiber cross-sectional area (CSA) and contractile function (Figure 2). The localized overexpression of miR-29c induced a significant increase in CSA (12%) as early as 7 days after electrotransfer (Figure 2A–C). Histologically, fiber polygonal shape was preserved as observed by HE staining and dystrophin labeling. Nuclei were localized in the periphery of the fibers in the miR-29c group, similarly to controls (Figure 2A). Moreover, maximal strength was increased by miR-29c overexpression (20%), in line with the CSA improvement (Figure 2D). At 30 days after pCMVmiR-29c electrotransfer, CSA further improved significantly (33%), similar to previous reports [11]. In addition, we observed muscle fibers with regular polygonal shape and normal extracellular space. Interestingly, we frequently observed fibers with numerous centralized nuclei, as previously reported (Figure 2E–G) [11]. Maximal strength was also increased by miR-29c overexpression (37%) compared to controls (Figure 2H).

### 2.3. Impact of miR-29c Overexpression on the AKT-mTOR Pathway

**Figure 3:**
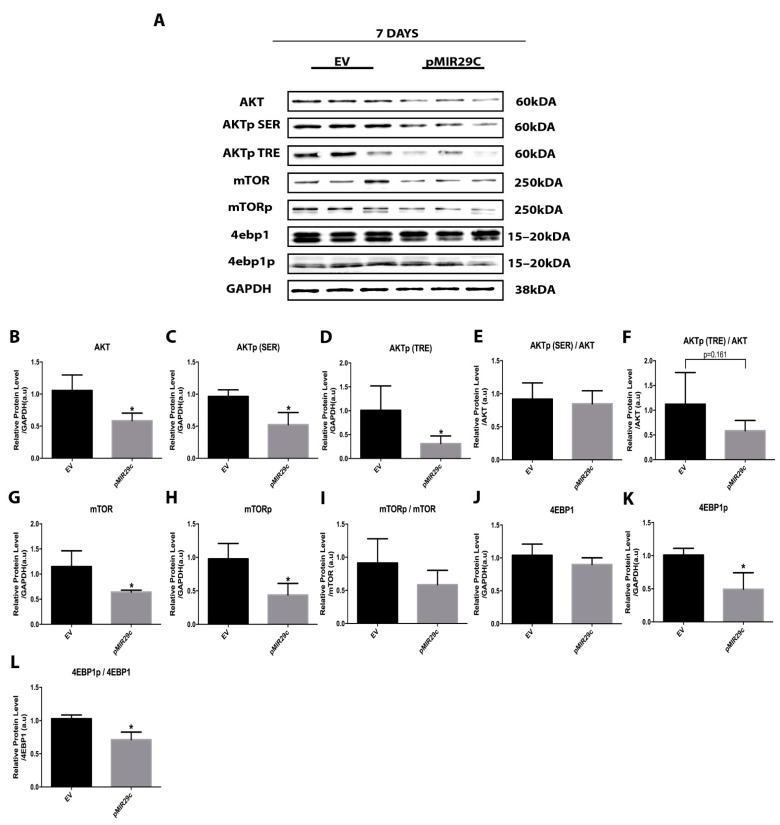
Phosphorylation and total protein involved in AKT/mTOR signaling after 7 days of miR-29c overexpression. (**A**) Representative bands after 7 days of miR-29c overexpression. Densitometry analysis of AKT (**B**–**F**), mTOR (**G**–**I**), and 4EBP1 (**J**–**L**) bands (*n* = 5 per group). Data are presented as mean ± SD. Statistical analysis included an unpaired *t*-test and a Mann–Whitney test. * *p* < 0.05 vs. EV group.

Next, we investigated whether the skeletal muscle hypertrophic state driven by miR-29c overexpression could impact the key anabolic AKT-mTOR signaling pathway. Interestingly, at 7 days after pMIR29c electrotransfer, we observed severe downregulation of total AKT (~50%) as well as the respective phosphorylated forms (AKTp (SER) ~50% and AKTp (TRE) ~70%). As total and phosphorylated forms were similarly downregulated, AKTp/AKT ratios were unaltered (Figure 3). Similarly, the expressions of total mTOR and its phosphorylated (pSer) form were decreased by ~45% and ~55%, respectively, after 7 days of miR-29c electrotransfer, leading to an unchanged mTORp/mTOR ratio (Figure 3). The downstream component 4EBP1 was downregulated (4EBP1p) by miR-29c by ~50%, leading to a reduced 4EBP1p/4EBP1 ratio (Figure 3).

**Figure 4:**
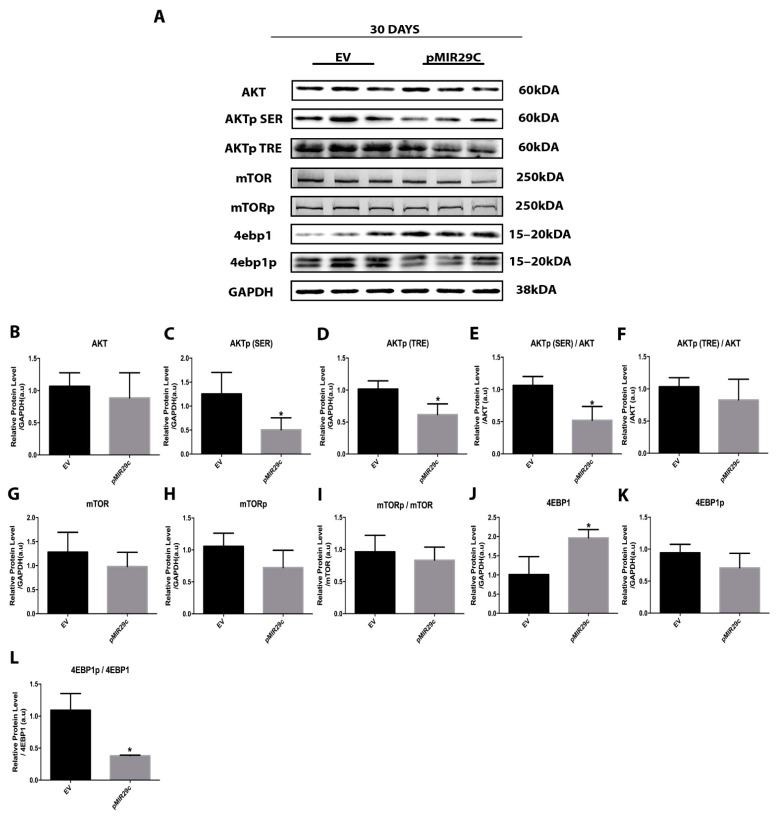
Phosphorylation and total protein involved in AKT/mTOR signaling after 30 days of miR-29c overexpression. (**A**) Representative bands after 30 days of miR-29c overexpression. Densitometry analysis of AKT (**B**–**F**), mTOR (**G**–**I**), and 4EBP1 (**J**–**L**) (*n* = 5 per group). Data are presented as mean ± SD. Statistical analysis included an unpaired *t*-test and a Mann–Whitney test. * *p* < 0.05 vs. EV group.

Since the miR29-c most pronounced hypertrophic effects occur on the 30th day post electrotransfer, we also investigated whether the AKT/mTOR pathway remained decreased for a longer term. Although the phosphorylated forms of mTOR and 4EBP1 were unaltered after 30 days of miR-29c electrotransfer as compared with control, the phosphorylated fractions of AKT remained downregulated (AKTp (SER) ~60% and AKTp (TRE) ~40%) (Figure 4). In addition, the total 4EBP1 content was increased by ~50% after 30 days of miR-29c electrotransfer, leading to a reduced 4EBP1p/4EBP1 ratio (Figure 4).

### 2.4. Impact of miR-29c Overexpression on Protein Synthesis

**Figure 5:**
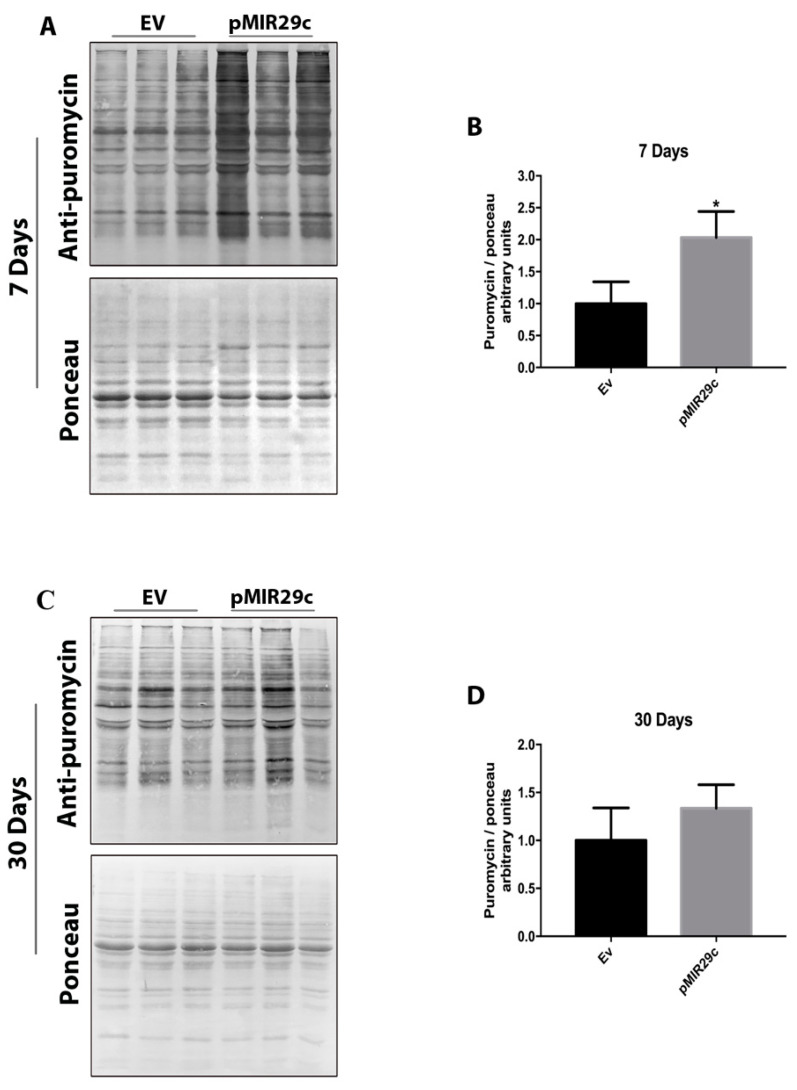
Protein synthesis analysis after 7 and 30 days of miR-29c overexpression. (**A**,**C**) Representative bands after 7 and 30 days of miR-29c overexpression in TA mouse skeletal muscle. (**B**,**D**) Densitometry analysis adjusted to the respective ponceau groups (*n* = 5 per group). Data are presented as mean ± SD. Statistical analysis included an unpaired *t*-test. * *p* < 0.05 vs. EV group.

Considering our previous results herein, showing a clear deactivation of the classical anabolic pathway AKT/mTOR by miR-29c overexpression, next, we decided to examine the overall levels of protein synthesis. Surprisingly, overall protein synthesis was strongly elevated (~2 fold) at 7 days of miR-29c overexpression, returning to normal levels on the 30th day (Figure 5).

## 3. Discussion

The purpose of this work was to get a deeper understanding of the mechanisms behind the hypertrophic effects of miR-29c in skeletal muscle. In spite of the fact that the AKT/mTOR pathway is inhibited by miR-29c overexpression, total protein synthesis is significantly increased, as shown herein.

In this study, we confirmed that pCMVmiR-29c electrotransfer imparted approximately 70% efficiency, a level sufficient to stimulate morphological and functional responses in skeletal muscle. These results demonstrate that the experimental paradigm adopted in this study was properly implemented.

Overexpression of miR-29c did not significantly affect fundamental tissue structure, as seen by the polygonal form and extracellular matrix of the fibers. In contrast, we observed a rise in the size of skeletal muscle fibers as early as 7 days after electrotransfer, and interestingly, by 30 days after electrotransfer, multiple centralized nuclei were revealed. These findings are consistent with prior research indicating that miR-29c overexpression activates satellite cells [11], resulting in the hypertrophic accretion of new nuclei to skeletal muscle fibers. How satellite cells are activated by miR-29c overexpression is an important molecular issue that will need to be explored in the future. One hypothesis considers that the increase of this microRNA in satellite cells may be sufficient to initiate the fiber hypertrophy process. In support of this hypothesis, we should note that prior research [11] indicated that treatment of developing C2C12 cells with the miR-29c mimic sequence is sufficient to induce a strong increase in myotube size. Importantly, the increases in size brought about by miR-29c overexpression were accompanied by increases in peak force.

Previous research suggested that the overexpression of miR-29c exerts at least a portion of its effects by inhibiting proteolytic-related pathways, particularly the ubiquitin-proteasome system, as well as decreasing the expression of important ubiquitin ligases such as MuRF1, Atrogin1, and HDAC4 [11]. These findings imply that miR-29c can inhibit basal myofibrillar breakdown, altering the usual equilibrium between proteolysis and protein synthesis in favor of myofibrillar protein accumulation. Mechanistically, herein, we provide additional insight by demonstrating that the major protein synthesis pathway, AKT/mTOR, is actually severely suppressed in skeletal muscle overexpressing miR-29c, thereby raising the possibility that the fiber enlargement induced by miR-29c may be the result of a simultaneous decrease in protein degradation and protein synthesis, with a positive balance over protein accumulation. On the other hand, we identified a large increase in global protein synthesis in muscles overexpressing miR-29c, around 2-fold, which is equivalent to strong responses in challenged skeletal muscle, such as the acute reaction to resistance exercise (about 3-fold) [13]. In light of these findings, we hypothesize that alternate pathways are triggered in miR-29c-overexpressing muscle, hence enhancing protein synthesis. It will be beneficial for future research to detail these pathways. Possible candidates include Calcineurin [14], Wnt/B-Catenin [15], B-adrenergic signaling [16], Nitric oxide, and PGC-alpha4 [17,18].

**Figure 6:**
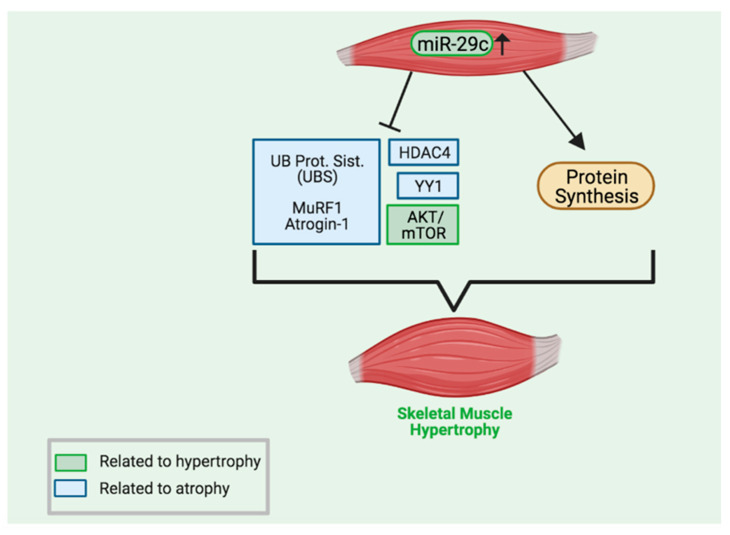
A summary of the pathways that are known to be impacted by high levels of miR-29c. MiR-29c-inhibited pathways are shown on the left (blue rectangles represent atrophy pathways and green rectangle relate to hypertrophic pathways). Please refer to [11,19] for more information. The present work shows that, despite the considerable increase in protein synthesis, components of the AKT/mTOR pathway are downregulated. The main results of miR-29c skeletal muscle overexpression are increased force and skeletal muscle development (for details see [11]). Created with BioRender.com.

Although our data hint at alternative anabolic pathways as potential targets of this miR-29c, it is crucial to highlight that the AKT/mTOR pathway is vast, and future research will focus on other components of this pathway in muscles that overexpress miR-29c. In Figure 6, we summarize the main signaling pathways regulated by miR-29c.

Overall, these results indicate that manipulation of miR-29c levels in skeletal muscle could be considered a potential strategy to either promote the increased performance or combat skeletal muscle deficiencies, such as those associated with aging, limb immobilization, corticosteroid therapy, and cachexia.

## 4. Materials and Methods

### 4.1. Experimental Animals

A total of 45 C57BL/6 male mice (~8 weeks old) were randomly divided into 5 groups: EV group for 7 and 30 days (animals that received empty vector pCMV-MIR), pMIR29C group for 7 and 30 days (animals that received miR-29c expression vector pCMVmiR-29c), and PBS group for 7 days (animals that received phosphate-buffered saline). The animals were kept in standard cages in a controlled environment (24 °C ± 1 °C, 12-h light-dark cycle) with free access to standard food and water. After 7 and 30 days from the electrotransfer, the animals were euthanized, and the tibialis anterior muscle was removed and stored at −80 °C for tissue and protein expression analysis.

### 4.2. Muscle Transfection by Eletrotransfer

The experiments involving transfection of the miR-29c expression vector were conducted on mouse tibialis anterior (TA) muscles, as described previously [11]. For the electroporation procedure, animals were anesthetized by a xylazine/ketamine cocktail (100 and 10 mg/kg, respectively). Then, hyaluronidase (40U, Sigma #H3506, St. Louis, MO, USA) was applied directly to TA muscles. After 30 min, muscles were injected with 20 µL of the miR-29c expression vector (2.5 µg/µL, pCMVmiR-29c, Origen, Rockville, MD, USA) or the empty vector (EV) pCMV-MIR or saline (PBS). Finally, electrodes were placed parallel along the muscle, and an electric pulse generator delivered a burst of pulses.

### 4.3. TA Morphometric Analysis

The TA muscle samples were sectioned in a cryostat (Leica CM1850 UV, Wetzlar, Germany). Immunofluorescence on 10-µm sections detected Dystrophin (Santa Cruz, rabbit anti- Dystrophin #sc-15376) and nuclei (by DAPI, 4′,6-diamidino-2-phenylindole). Photomicrographs were acquired on Axio Scope.A1 (Carl Zeiss Microscopy GmbH, Göttingen, Germany). The muscle fibers’ cross-sectional area (CSA) was measured using the ImageJ software (v. 1.45s, National Institutes of Health, Bethesda, MD, USA). Around 800–1200 fibers were analyzed per group. The overall integrity and architecture of the tissue were evaluated by hematoxylin-eosin stains.

### 4.4. Puromycin Assay

Overall de novo TA muscle protein synthesis was determined by the SUnSET method [20]. Animals were anesthetized as described previously. Then, puromycin solution was injected intraperitoneally (i.p.) into animals (0.040 µmol/g body weight). Muscles were removed at exactly 30 min after injections and snap-frozen in liquid nitrogen for further western blotting analysis using an anti-puromycin antibody (Cat # MABE343, Merck Millipore, Burlington, MA, USA).

### 4.5. Maximal Strength Test (1RM)

The 1RM test (i.e., strength during one maximal repetition) was performed as previously described. Briefly, an 8V stimulus was applied in a TA motor point, and a load (~8% of body weight) was fixed in the distal portion of the hind paw. The animals typically performed ankle dorsiflexion with one or two maximum contractions before fatigue [21].

### 4.6. Western Blotting

Samples were powdered in a liquid nitrogen-chilled mortar and homogenized in RIPA buffer (1 mM EDTA, pH 7.4, 0.0625% sodium deoxycholate, 0.0625% nonidet P-40, 6.2 mM sodium phosphate, and protease and phosphatase inhibitor cocktail—Thermo Scientific cat#78445). After 30 min incubation on ice, the homogenates were centrifuged (10,000 rpm for 10 min at 4 °C). Protein levels in supernatants were measured by Bradford assay to normalize gel loadings.

Total protein (25 µg) was run in 12% polyacrylamide gels (60–120 V for 60 min–120 min) and blotted to a polyvinylidene difluoride membrane (0.45 µm, ThermoFisher, CAT: 88518, Rockford, IL, USA) through semi-dry transfer (20 V for 120 min). Membranes were stained by Ponceau S to attest consistent loading. After that, membranes were incubated in blocking solution (5% BSA in Tris-buffered saline solution–TBST: 0.5 M NaCl, 50 mM Tris-HCl pH 7.4, 0.1% Tween 20) at room temperature for 1 hour, washed in TBST (3 rounds of 5 min) and further incubated in primary antibody solution overnight at 4 °C. Primary antibodies were as follows: rabbit anti-GAPDH (1:3000; Cell Signaling, #2118, Danvers, MA, USA); rabbit anti-AKT (1:1000; Cell Signaling, #9272); rabbit anti-AKTp (TRE) (1:1000; Cell Signaling, #4056); rabbit anti-AKTp (SER) (1:1000; Cell Signaling, #4058s); rabbit anti-mTOR (1:1000; Cell Signaling, #2972s); rabbit anti-pmTOR Ser (1:1000; Cell Signaling, #2971s); rabbit anti-4EBP1 (1:1000; Cell Signaling, #9644); and rabbit anti-4EBP1p (TRE) (1:1000; Cell Signaling, #9459s).

Membranes were washed in TBST (3 rounds of 5 min) to remove primary antibodies and treated with secondary antibody solution (1:30,000, goat anti-rabbit peroxidase cat#111035003, Jackson ImmunoResearch; incubation for 1 hour at room temperature). Then, membranes were washed again in TBST (3 × 5 min). Bound proteins were visualized by the Fusion FX5 XT Vilber Loyurmat imaging system, using 2 min of Luminata^TM^ incubation (cat#WBLUF0500, Millipore, Burlington, MA, USA). GADPH protein levels were determined as housekeeping control for normalization.

### 4.7. RNA Extraction and Real-Time PCR

Total RNA was extracted from muscle samples (25 mg) using Trizol (Invitrogen) according to the manufacturer’s instructions. The samples were dissolved in ultrapure water, and their concentrations were measured using a spectrophotometer (Eppendorf) by absorbance at 260 nm. The integrity of the RNA was verified on a denaturant 1 percent agarose ethidium bromide-stained gel, and the purity of the RNA was confirmed by establishing the ratio between measurements at 260 and 280 nm.

For miR expression analysis, 10 ng of the total RNA was used in a cDNA reaction containing 5X Loop Primers for RT-PCR specific for each miRNA that was analyzed, 100 nM dNTP mix with dTTP, 10× RT buffer, RNase inhibitor (20 U/uL), nuclease-free water, and 50 units of MultiScribe™ RT enzyme (TaqMan^®^ microRNA RT Kit—BTM) performed at 16 °C for 30 min followed by 42 °C for 30 and 5 min at 85 °C. One microliter of cDNA was used in real-time PCR, containing TaqMan^®^ Universal Master Mix II (ABTM), TaqMan^®^ MGB probes specific for the analyzed miR (TaqMan^®^ microRNA Assays—ABTM cat#4427975), and nuclease-free water. The cycle settings were 50 °C for 2 min, 95 °C for 10 min, followed by 40 cycles of 95 °C for 15 s, and 60 °C for 1 min. Fluorescence intensity was quantified with a qPCR thermocycler (Corbett RotorGene 6000, Qiagen©, Hilden, Germany).

### 4.8. Statistical Analysis

Data analysis was performed in GraphPad Prism 7.0. Multiple comparisons were established using either one-way ANOVA followed by Tukey’s posthoc test (for parametric data) or the Kruskal–Wallis test of one-way ANOVA followed by Dunn’s posthoc test (for non-parametric data). The unpaired *t*-test was employed to compare the differences between two groups when the data were parametric, or the Mann–Whitney test when the data were non-parametric. Data were presented as mean ± SD or SEM, and a statistical significance of *p* < 0.05 was considered.

## 5. Conclusions

Our results suggest that miR-29c overexpression drives increasing overall protein synthesis without activating key components of the AKT-mTOR pathway.

## Data Availability

The data that support the findings of this study are available from the corresponding author, [ASM], upon reasonable request.
